# Icanbelimod (CBP-307), a next-generation Sphingosine-1-phosphate receptor modulator, in healthy men: pharmacokinetics, pharmacodynamics, safety, and tolerability in a randomized trial in Australia

**DOI:** 10.3389/fimmu.2024.1380975

**Published:** 2024-06-17

**Authors:** Jason Lickliter, Xin Yang, Jiawang Guo, Wubin Pan, Zheng Wei

**Affiliations:** ^1^ Nucleus Network, Melbourne, VIC, Australia; ^2^ Suzhou Connect Biopharmaceuticals, Ltd, Taicang, China; ^3^ Connect Biopharma, San Diego, CA, United States

**Keywords:** Sphingosine-1-phosphate receptor modulator, icanbelimod, CBP-307, pharmacokinetics, pharmacodynamics, safety, lymphocyte reduction

## Abstract

**Background:**

Icanbelimod (formerly CBP-307) is a next-generation S1PR modulator, targeting S1PR_1_. In this first-in-human study, icanbelimod was investigated in healthy men in Australia.

**Methods:**

Participants were randomized 3:1, double-blind, to icanbelimod or placebo in four single-dose cohorts (0.1 mg, 0.25 mg, 0.5 mg [n=8 per cohort], 2.5 mg [n=4]) or for 28-days once-daily treatment in two cohorts (0.15 mg, 0.25 mg [n=8 per cohort]). Participants in the 0.25-mg cohort received 0.1 mg on Day 1. Treatments were administered orally after fasting; following one-week washout, icanbelimod was administered after breakfast in the 0.5-mg cohort.

**Results:**

Icanbelimod exposure increased rapidly and dose-dependently with single and multiple dosing (T_max_ 4–7 hours). Lymphocyte counts decreased rapidly after single (-11%, 0.1 mg; -40%, 0.25 mg; -71%, 0.5 mg; -77%, 2.5 mg) and multiple doses (-49%, 0.15 mg; -75%, 0.25 mg), and recovered quickly, 7 days after dosing. After single-dose 0.5 mg, although a high-fat breakfast versus fasting did not affect maximal decrease, lymphocyte counts tended to be lower after breakfast across most timepoints up to 72 hours. Twenty-eight participants (63.6%) experienced mainly mild treatment-emergent adverse events (TEAEs). After single-dose icanbelimod, the most common TEAEs were headache (28.6%, n=6) and dizziness (19.0%, n=4). Three participants experienced transient bradycardia, with one serious, following single-dose 2.5 mg icanbelimod. After multiple-dose icanbelimod, the most common TEAEs were headache (50.0%, n=6) and lymphopenia (41.7%, n=5), and two participants withdrew due to non-serious TEAEs. Up-titration attenuated heart rate reductions.

**Conclusion:**

Icanbelimod was well-tolerated up to 0.5 mg and effectively reduced lymphocyte counts.

**Clinical trial registration:**

ClinicalTrials.gov, identifier NCT02280434.b

## 1 Introduction

Autoimmune diseases affect a large proportion of the global population and can be extremely debilitating; examples include inflammatory bowel disease (IBD), multiple sclerosis (MS), rheumatoid arthritis, and psoriasis (1–3). IBD comprises both ulcerative colitis (UC) and Crohn’s disease. Between 1990 and 2017 the global prevalence of IBD increased substantially and, in 2015, an estimated 3.1 million US adults had received a diagnosis (4, 5). Current treatments for autoimmune diseases, such as IBD, are associated with ineffectiveness (30–60% of patients with UC experience remission in clinical practice, often resulting in surgery) and safety issues may limit their use (6–12). For instance, glucocorticoids are associated with opportunistic infection, osteoporosis and impaired glucose tolerance, Janus kinase inhibitors may increase the risks of major cardiovascular events, and anti-TNF-α therapy has been linked with opportunistic infection and cancer (6–12).

Sphingosine-1-phosphate receptor (S1PR) modulators inhibit egress of lymphocytes from lymph nodes (13). The first-in-class S1PR modulator, fingolimod, gained approval for the treatment of MS in the US and Europe over a decade ago. However, fingolimod demonstrates non-selective activity towards S1PR subtypes, potentially associated with treatment-limiting bradycardia and hypertension (14–16). Lymphocyte counts also remain low for at least 4 weeks after discontinuation of fingolimod, compatible with a long elimination half-life of ~8 days (17, 18), potentially posing issues with opportunistic infections (19, 20). To minimize these effects, next-generation S1PR modulators selectively target S1PR_1,5_ (ozanimod and siponimod), S1PR_1,4,5_ (etrasimod), S1PR_1_ (ponesimod) and, in general, have shorter elimination half-lives than fingolimod (21–27). So far, the next-generation S1PR modulators ozanimod, siponimod, and ponesimod are approved in the US and Europe for the treatment of MS, and ozanimod is also licensed for the treatment of UC.

Icanbelimod (formerly known as CBP-307, with the chemical name 1-(2-fluoro-4-(5-(4-isobutylphenyl)-1, 2, 4-oxadiazol-3-yl) benzyl)azetidine-3-carboxylic acid hemihydrate) is a next-generation, highly potent S1PR_1_ agonist with no notable activity for S1PR_3_ in Chinese hamster ovary cells expressing human S1PRs. *In vivo* animal studies demonstrated large (>50%) reductions in lymphocyte counts and, notably, a short elimination half-life (5.3 hours in rats) when indirectly compared with fingolimod (23.4 hours) (28). Rapid drug elimination, allowing particularly fast recovery of lymphocyte counts (typically observed within 12–48 hours after discontinuation of icanbelimod in animal studies), should reduce the risk of opportunistic infections and, if required to treat disease flare-ups, allow rapid switching to alternative therapies (29).

Here we report the first-in-human study of icanbelimod, evaluating the safety, tolerability, pharmacokinetics (PK) and pharmacodynamics (PD) of icanbelimod following oral administration of single and multiple ascending doses in healthy men in Australia. Another Phase 1 study has also been completed in healthy men in China, and icanbelimod is being investigated in a Phase 2 trial of patients with moderate-to-severe UC.

## 2 Methods

### 2.1 Study design

This randomized, double-blind, placebo-controlled, parallel-group, dose escalation study (NCT02280434) of orally administered icanbelimod in healthy men at a single site in Australia. The first participant was enrolled in November 2014 and the study was completed in August 2015.

**Figure 1:**
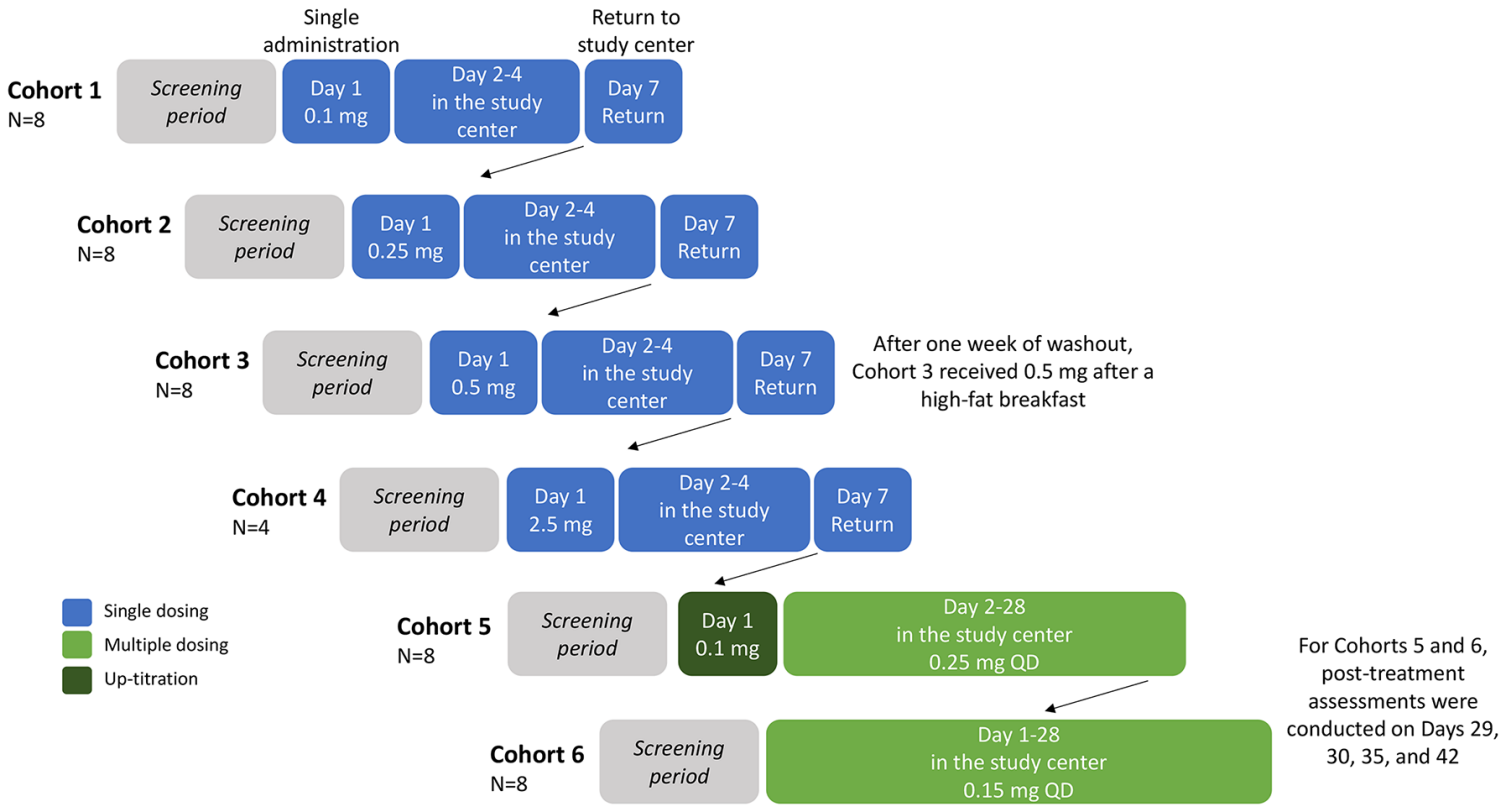
Study design. Participants were randomized 3:1, double-blind, per cohort to orally receive icanbelimod or placebo (with approximately 240 mL of non-carbonated water), all administered in the study center. All participants were required to fast from 10 hours predose to 4 hours postdose and all patients received standardized meals whilst in the study center. Cohort 3 participants returned after one week of washout, for an additional 0.5 mg dose administered 30 minutes after a high-fat breakfast (preceded by fasting overnight and for 4 hours postdose). Participants in Cohorts 1–4 were discharged from the study center on Day 4, and returned for final assessments on Day 7. QD, once daily.

The study consisted of single and multiple ascending dose stages (**Figure 1**). In both stages, all participants were required to fast from 10 hours predose to 4 hours postdose and all patients received standardized meals whilst in the study center.

In the single ascending dose stage, 28 participants were enrolled into four icanbelimod dose cohorts (0.1 mg, 0.25 mg, 0.5 mg, 2.5 mg) (**Figure 1**). For the three lowest doses (0.1 mg, 0.25 mg, 0.5 mg), 8 participants were randomized 3:1 per cohort to receive one capsule of icanbelimod or matching placebo. In the other cohort (2.5 mg), 4 participants were randomized 3:1 to receive one capsule of icanbelimod or matching placebo. Dosing cohorts started sequentially, beginning with the lowest dose, escalating to the next dose after one week of satisfactory safety assessments. In each cohort, one participant received icanbelimod and one participant received placebo 24 hours before the remaining participants commenced treatment. A food effect was evaluated in participants in the 0.5 mg cohort; one week after receiving 0.5 mg icanbelimod in the fasted state, the same participants received 0.5 mg icanbelimod 30 minutes after a standardized high-fat breakfast (preceded by fasting overnight and for 4 hours postdose).

In the multiple ascending dose stage, 16 participants were enrolled into two icanbelimod dose cohorts (0.15 mg, 0.25 mg); 8 participants were randomized 3:1 per cohort to receive one capsule of icanbelimod or matching placebo once-daily (QD) for 28 days (**Figure 1**). In the icanbelimod 0.25 mg cohort, participants received a lower first dose (0.1 mg) on Day 1, followed by 0.25 mg QD, to reduce the potential first-dose effect on heart rate.

For this first-in-human study, icanbelimod dose selection was based on preclinical findings. The lowest dose was selected on the basis that the minimal efficacious dose in rats (0.01 mg/kg when reducing peripheral blood lymphocyte counts) corresponds to 0.12 mg for a 70-kg person. The no-observed-adverse-effect level (NOAEL) for rats (1 mg/kg) and dogs (1 mg/kg) from 28-day repeat-dose toxicology studies correspond to 11.2 mg and 35 mg, respectively, for a 70-kg person. Therefore, in the current study, all doses were considerably lower than the NOAEL.

### 2.2 Ethics statement

This study was performed in accordance with Good Clinical Practice guidelines and the Declaration of Helsinki. The clinical study protocol, informed consent document, and all other relevant study documentation were approved by the Independent Ethics Committee at Alfred Hospital (Melbourne, Australia). All participants provided their written informed consent before study commencement.

### 2.3 Participants

All participants were healthy men with a body mass index (BMI) between 19 and 30 kg/m^2^, heart rate ≥55 bpm, no significant medical history and normal renal, cardiac and pulmonary functions (**Supplementary Table 1**).

### 2.4 Randomization and blinding

Participants were assigned a randomization code at screening to determine the study treatment dispensed to each participant. The sponsor, investigators, nurses, and participants remained blinded to the assigned study treatment at all times. Unblinding was allowed only if the medical monitor and investigator believed it was necessary in order to treat an adverse event (AE) or in the case of an emergency.

### 2.5 Pharmacokinetic and pharmacodynamic assessments

PK parameters were icanbelimod plasma concentrations, AUC_last_, AUC_inf_, C_max_, T_max_, t_1/2_. PD parameters were absolute lymphocyte count analysis. Timepoints for PK and PD blood collection are described in **Supplementary Table 2**. For PK assessment, 5 mL blood samples were drawn from the arm contralaterally. For PD analysis, 3 mL blood samples were collected into K2-EDTA vacutainers. The PD samples were directly used for hematology analysis following the standard operation procedure of the clinical center.

### 2.6 Safety assessments

Safety assessments included AE monitoring, hematology, clinical chemistry, urinalysis, serology, and other tests (alcohol breath, urine drug screening, tuberculosis, pulmonary function), vital signs, 12-lead ECG, Holter monitoring, physical examination and, in the multiple dosing stage, chest X-ray and ophthalmologic). All AEs were coded using the Medical Dictionary for Regulatory Activities version 11.1.

Safety was assessed throughout the study. In the single ascending dose stage, participants remained in the clinical center for safety assessments pre-treatment (Day -1), on the day of treatment (Day 1) and daily post-treatment (discharged on Day 4), returning to the clinic for final safety assessment on Day 7. In the multiple ascending dose stage, participants remained in the clinical center for daily safety assessments pre-treatment (Day -1), throughout treatment (Days 1–28), and post-treatment (discharged on Day 30), with further safety assessments on Days 35 and 42.

### 2.7 Analysis sets

Both the PD and safety sets included all participants who received at least one dose of study drug. The PK set included all participants with at least one PK parameter that could be adequately estimated.

### 2.8 Statistical analysis

The sample size was based on accepted standards for this type of investigation at the time of study; no formal sample size calculations were performed for this first-in-human study.

Descriptive statistics were used for demographic, safety, ECG, PK and PD data. PK parameters were calculated using noncompartmental methods using WinNonLin Version 6.3. Calculation of AUC_last_ and AUC_inf_ were conducted using the linear trapezoidal method. The terminal elimination rate constant λz [1/h] was calculated by log-linear regression of the terminal segment of the baseline-corrected plasma concentration versus time curve. The apparent terminal elimination half-life, t_1/2_, was calculated as ln(2)/λz.

## 3 Results

### 3.1 Participant demographics

All 44 participants were healthy men (**Supplementary Table 3**). Mean (SD) age was 25.9 (5.14) years and BMI 23.9 (2.24) kg/m^2^. All participants were Caucasian, except for two Asian men and two other ethnic origin. In each of the single ascending dose cohorts (0.1 mg, 0.25 mg, 0.5 mg, 2.5 mg) and multiple ascending dose cohorts (0.15 mg, 0.25 mg QD), eight participants received icanbelimod (n=6) or placebo (n=2), except for the highest (2.5 mg) single dose cohort (icanbelimod, n=3; placebo, n=1). Forty-two out of the 44 participants completed the study per protocol (**Supplementary Figure 1**).

### 3.2 Pharmacokinetics

**Figure 2:**
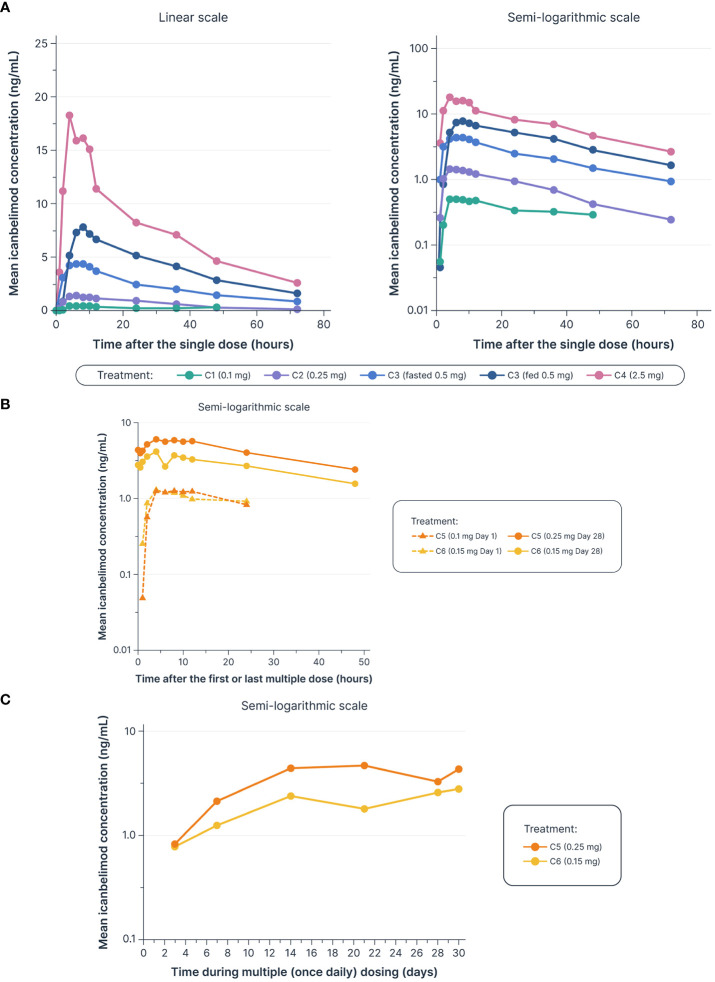
Dose-dependent increases were observed in mean icanbelimod concentration after **(A)** single dosing^a–c^ **(B)** and multiple (once daily) dosing,^c–e^ **(C)** with C_trough_ reaching steady state by Day 14 of multiple dosing^c–e. a^In Cohorts 1 (C1), 2 (C2), and 3 (C3), participants were randomized to single dose icanbelimod 0.1 mg, 0.25 mg, and 0.5 mg (n=6), respectively, or placebo (n=2). ^b^In Cohort 4 (C4), participants were randomized to single dose icanbelimod 2.5 mg (n=4) or placebo (n=1). ^c^All treatments were administered after fasting, except for C3 participants who received a 0.5 mg dose after fasting and returned after one week of washout for an additional 0.5 mg dose administered following a high-fat breakfast. ^d^In Cohort 5 (C5), participants were randomized to icanbelimod 0.1 mg for one day, followed by 0.25 mg once daily (n=6), or placebo (n=2). ^e^In Cohort 6 (C6), participants were randomized to icanbelimod 0.15 mg once daily (n=6) or placebo (n=2).

Dose-dependent increases in AUC_last_ and C_max_ were observed with single and multiple dosing (**Table 1**, **Figure 2**). Mean T_max_ ranged from 5.0–7.3 hours following single dosing. Icanbelimod followed mono-exponential elimination, with a mean terminal t_1/2_ of 25.2 hours. In the single-dose 0.5 mg icanbelimod group, in the fasted state, mean T_max_ was 5.0 hours (**Table 1**). In the same participants one week later, single-dose 0.5 mg icanbelimod following a high-fat breakfast delayed T_max_ to 10.7 hours, and AUC_last_ and C_max_ increased by 1.81-fold and 1.77-fold, respectively. During the 28-day multiple-dose phase, plasma concentration of icanbelimod reached steady state on Day 14 (**Figure 2**). Mean T_max_ ranged from 4.3–6.8 hours (**Table 1**).

**Table 1:** Summary of icanbelimod PK parameters after single and multiple (once daily) dosing^a^.

Single dosing	Cohort 1,^b^ 0.1 mg	Cohort 2,^b^ 0.25 mg		Cohort 3,^b^ 0.5 mg (fasted)		Cohort 3,^b^ 0.5 mg (fed)	Cohort 4,^c^ 2.5 mg
AUC_last_ (ng*h/mL)	13.4 ± 3.54 (n=6)	45.1 ± 5.25 (n=6)		160 ± 22.8 (n=6)		290 ± 28.1 (n=6)	550 ± 96.3 (n=3)
AUC_inf_ (ng*h/mL)	Not determinable^d^	63.7 ± 2.14 (n=3)		214 ± 58.3 (n=3)		355 ± 44.3 (n=5)	710 ± 164 (n=2)
C_max_	0.537 ± 0.0931	1.53 ± 0.0935		4.84 ± 0.706		8.58 ± 1.11	19.0 ± 3.55
(ng/mL)	(n=6)	(n=6)		(n=6)		(n=6)	(n=3)
T_max_ (h)	7.33 ± 1.33 (n=6)	5.33 ± 0.99 (n=6)		5.00 ± 0.86 (n=6)		10.67 ± 2.72 (n=6)	6.00 ± 2.00 (n=3)
Lambda (1/h)	Not determinable^d^	0.0300 ± 0.00215 (n=3)		0.0242 ± 0.00112 (n=3)		0.0268 ± 0.00113 (n=5)	0.0314 ± 0.00544 (n=2)
T_1/2_ (h)	Not determinable^d^	23.3 ± 1.70 (n=3)		28.8 ± 1.28 (n=3)		26.0 ± 1.17 (n=5)	22.8 ± 3.96 (n=2)
DN AUC_last_ (ng*h/mL)	134 ± 35.4 (n=6)	180 ± 21.0 (n=6)		320 ± 45.6 (n=6)		579 ± 56.2 (n=6)	220 ± 38.5 (n=3)
DN AUC_inf_ (ng*h/mL)	Not determinable^d^	255 ± 8.58 (n=3)		427 ± 117 (n=3)		710 ± 88.6 (n=5)	284 ± 65.6 (n=2)
DN C_max_ (ng/mL)	5.37 ± 0.931 (n=6)	6.12 ± 0.374 (n=6)		9.68 ± 1.41 (n=6)		17.2 ± 2.22 (n=6)	7.60 ± 1.42 (n=3)
Multiple dosing	Cohort 5^e^				Cohort 6^f^		
	Day 1, 0.1 mg		Day 28, 0.25 mg		Day 1, 0.15 mg		Day 28, 0.15 mg
AUC_0-24 h_ (ng*h/mL)	24.5 ± 3.26 (n=5)		125 ± 12.4 (n=4)		26.3 ± 7.45 (n=5)		79.7 ± 27.3 (n=6)
AUC_last_ (ng*h/mL)	–		203 ± 21.9 (n=4)		–		131 ± 44.7 (n=6)
AUC_inf_ (ng*h/mL)	Not determinable^d^		Not determinable^d^		Not determinable^d^		Not determinable^d^
C_max_ (ng/mL)	1.45 ± 0.214 (n=5)		6.30 ± 0.588 (n=4)		1.32 ± 0.422 (n=6)		4.23 ± 1.45 (n=6)
T_max_ (h)	6.80 ± 1.50 (n=5)		6.50 ± 1.50 (n=4)		5.00 ± 0.68 (n=6)		4.33 ± 0.33 (n=6)
Lambda (1/h)	Not determinable^d^		Not determinable^d^		Not determinable^d^		Not determinable^d^
T_1/2_ (h)	Not determinable^d^		Not determinable^d^		Not determinable^d^		Not determinable^d^
DN AUC_0-24_ h (ng*h/mL)	245 ± 32.6 (n=5)		499 ± 49.5 (n=4)		175 ± 49.7 (n=5)		531 ± 182 (n=5)
DN AUC_last_ (ng*h/mL)	–		812 ± 87.5 (n=4)		–		872 ± 298 (n=6)
DN AUC_inf_ (ng*h/mL)	Not determinable^d^		Not determinable^d^		Not determinable^d^		Not determinable^d^
DN C_max_ (ng/mL)	14.5 ± 2.14 (n=5)		25.2 ± 2.35 (n=4)		8.79 ± 2.82 (n=6)		28.2 ± 9.66 (n=6)

All values are mean ± standard deviation.

a All treatments were administered after fasting, except for Cohort 3 participants who received a 0.5 mg dose after fasting and returned after one week of washout for an additional 0.5 mg dose administered following a high-fat breakfast.

b In Cohorts 1, 2, and 3, participants were randomized to single dose icanbelimod 0.1 mg, 0.25 mg, and 0.5 mg (n=6), respectively, or placebo (n=2).

c In Cohort 4, participants were randomized to single dose icanbelimod 2.5 mg (n=4) or placebo (n=1).

d Not determinable because the terminal elimination rate constant for plasma concentration versus time profiles could not be reliably estimated for all participants.

e In Cohort 5, participants were randomized to icanbelimod 0.1 mg for one day, followed by 0.25 mg once daily (n=6), or placebo (n=2).

f In Cohort 6, participants were randomized to icanbelimod 0.15 mg once daily (n=6) or placebo (n=2).

AUC_inf_, area under the curve from 0 h to infinity; AUC_last_, area under the curve from 0 h to the last time point on lower limit of detection; AUC_0-24_, area under the curve from 0 h to 24 h; C_max_, maximum plasma concentration; DN, dose normalized; h, hour; PK, pharmacokinetic; T_max_, time to maximum plasma concentration; t_1/2_, elimination half-life; Lambda, terminal elimination rate constant.

### 3.3 Pharmacodynamics

**Figure 3:**
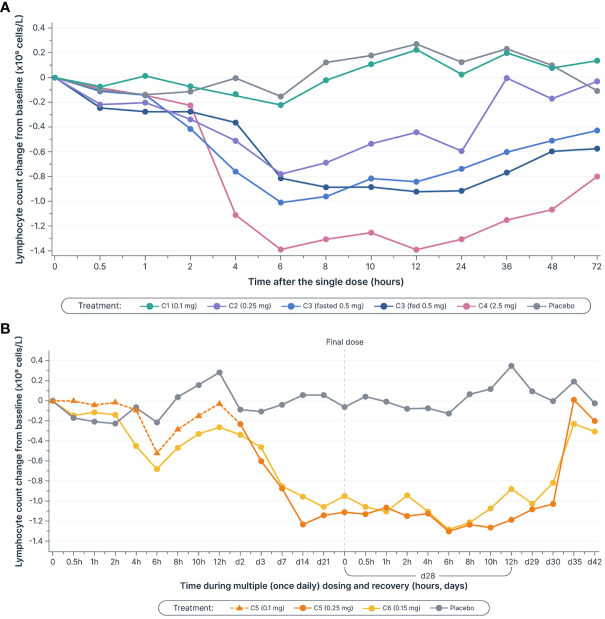
Mean lymphocyte counts decreased in a dose-dependent manner **(A)** after single doses^a–c^ **(B)** and multiple (once daily) doses of icanbelimod, reached steady state by Day 14, and returned to baseline levels one week after the final dose was administered^c–e^. Time is not shown uniformly in either panel. ^a^In Cohorts 1 (C1), 2 (C2), and 3 (C3), participants were randomized to single dose icanbelimod 0.1 mg, 0.25 mg, and 0.5 mg (n=6), respectively, or placebo (n=2). ^b^In Cohort 4 (C4), participants were randomized to single dose icanbelimod 2.5 mg (n=4) or placebo (n=1). ^c^All treatments were administered after fasting, except for C3 participants who received a 0.5 mg dose after fasting and returned after one week of washout for an additional 0.5 mg dose administered following a high-fat breakfast. ^d^In Cohort 5 (C5), participants were randomized to icanbelimod 0.1 mg for one day, followed by 0.25 mg once daily (n=6), or placebo (n=2). ^e^In Cohort 6 (C6), participants were randomized to icanbelimod 0.15 mg once daily (n=6) or placebo (n=2).

Single and multiple dose administration of icanbelimod resulted in rapid dose-dependent reductions in total circulating lymphocyte count (**Figure 3**). Following single-dose 0.1, 0.25, 0.5, and 2.5 mg icanbelimod, maximal mean decreases in lymphocyte count (approximately 6 hours post-dose) were 11%, 40%, 71%, and 77%, respectively. Although a high-fat breakfast versus fasting before receiving 0.5 mg icanbelimod did not affect maximal decrease, mean lymphocyte counts tended to be lower in the fed state across the majority of timepoints up to 72 hours. In the multiple-dose phase, lymphocyte counts reached steady state by Day 14, decreasing by 49% and 75% with icanbelimod 0.15 mg QD and 0.25 mg QD, respectively, and returned to baseline levels one week after the last dose was administered.

### 3.4 Safety

In the single-dosing phase, treatment-emergent adverse events (TEAEs) were reported for 12/21 (57.1%) participants treated with any dose of icanbelimod and 3/7 (42.8%) with placebo (**Table 2**) and, in the multiple-dose phase, for 11/12 (91.7%) and 2/4 (50.0%), respectively (**Table 3**).

**Table 2:** TEAEs with single dosing^a^.

	Placebo	Icanbelimod				
		0.1 mg	0.25 mg	0.5 mg	2.5 mg	Combined
	N=7	N=6	N=6	N=6	N=3	N=21
	n (%)	n (%)	n (%)	n (%)	n (%)	n (%)
Any TEAE	3 (42.8)	1 (16.7)	3 (50.0)	5 (83.3)	3 (100)	12 (57.1)
Mild	3 (42.8)	0	3 (50.0)	5 (83.3)	3 (100)	11 (52.4)
Moderate	0	1 (16.7)	0	0	3 (100)	4 (19.0)
Severe	0	0	0	0	1 (33.3)	1 (4.7)
Serious/significant	0	0	0	0	1 (33.3)	1 (4.7)
Death	0	0	0	0	0	0
Nervous system disorders	1 (14.3)	0	2 (33.3)	5 (83.3)	3 (100)	10 (47.6)
Headache	1 (14.3)	0	2 (33.3)	2 (33.3)	2 (66.7)	6 (28.6)
Dizziness	0	0	0	3 (50.0)	1 (33.3)	4 (19.0)
General disorders and administration site conditions	0	0	0	1 (16.7)	1 (33.3)	2 (9.5)
Fatigue	0	0	0	1 (16.7)	0	1 (4.7)
Flushing	0	0	0	0	1 (33.3)	1 (4.7)
gastrointestinal disorders	1 (14.3)	0	0	1 (16.7)	1 (33.3)	2 (9.5)
Abdominal pain	1 (14.3)	0	0	0	0	0
Flatulence	0	0	0	1 (16.7)	0	1 (4.7)
Diarrhea	0	0	0	0	1 (33.3)	1 (4.7)
Nausea	0	0	0	0	1 (33.3)	1 (4.7)
Metabolism and nutrition disorders	0	1 (16.7)	0	0	0	1 (4.7)
Hyperlipasemia	0	1 (16.7)	0	0	0	1 (4.7)
Infections and infestation	1 (14.3)	0	0	0	0	0
Nasopharyngitis	1 (14.3)	0	0	0	0	0
Cardiac disorders	0	0	0	0	3 (100)	3 (14.3)
Bradycardia	0	0	0	0	1 (33.3)	1 (4.7)
Sinus bradycardia	0	0	0	0	2 (66.7)	2 (9.5)
Transient asystole	0	0	0	0	1 (33.3)	1 (4.7)

a All treatments were administered after fasting, except for participants who received a 0.5 mg dose after fasting and returned after one week of washout for an additional 0.5 mg dose administered following a high-fat breakfast.

**Table 3:** TEAEs with multiple (once daily) dosing^a^.

	Placebo	Icanbelimod		
		0.15 mg	0.25 mg^b^	Combined
	N=4	N=6	N=6	N=12
	n (%)	n (%)	n (%)	n (%)
Any TEAE	2 (50.0)	5 (83.3)	6 (100)	11 (91.7)
Mild	2 (50.0)	5 (83.3)	5 (83.3)	10 (83.3)
Moderate	0	1 (16.7)	1 (16.7)	2 (16.7)
Severe	0	1 (16.7)	2 (33.3)	3 (25.0)
Serious/significant	0	0	0	0
Death	0	0	0	0
TEAEs causing study drug discontinuation	0	0	2 (33.3)	2 (16.7)
Transaminases increased	0	0	1 (16.7)	1 (8.3)
Atrioventricular block second degree	0	0	1 (16.7)	1 (8.3)
Nervous system disorders	1 (25.0)	4 (66.7)	2 (33.3)	6 (50.0)
Headache	1 (25.0)	4 (66.7)	2 (33.3)	6 (50.0)
Dizziness	0	0	1 (16.7)	1 (8.3)
General disorders and administration site conditions	0	1 (16.7)	2 (33.3)	3 (25.0)
Chest pain	0	0	1 (16.7)	1 (8.3)
Fatigue	0	1 (16.7)	1 (16.7)	2 (16.7)
gastrointestinal disorders	0	2 (33.3)	2 (33.3)	4 (33.3)
Abdominal pain	0	1 (16.7)	0	1 (8.3)
Diarrhea	0	0	1 (16.7)	1 (8.3)
Tongue ulceration	0	1 (16.7)	0	1 (8.3)
Gastroesophageal reflux disease	0	0	1 (16.7)	1 (8.3)
Nausea	0	1 (16.7)	1 (16.7)	2 (16.7)
Infections and infestation	0	0	1 (16.7)	1 (8.3)
Upper respiratory tract infection	0	0	1 (16.7)	1 (8.3)
Cardiac disorders	0	2 (33.3)	1 (16.7)	3 (25.0)
Sinus bradycardia	0	1 (16.7)	0	1 (8.3)
Extrasystole	0	1 (16.7)	0	1 (8.3)
Atrioventricular block second degree	0	0	1 (16.7)	1 (8.3)
Blood and lymphatic system disorders	0	1 (16.7)	4 (66.7)	5 (41.7)
Lymphopenia	0	1 (16.7)	4 (66.7)	5 (41.7)
Musculoskeletal and connective tissue disorders	1 (25.0)	2 (33.3)	2 (33.3)	4 (33.3)
Back pain	0	0	1 (16.7)	1 (8.3)
Musculoskeletal pain	1 (25.0)	1 (16.7)	1 (16.7)	2 (16.7)
Myofascial pain at neck	0	1 (16.7)	0	1 (8.3)
Respiratory, thoracic and mediastinal disorders	0	1 (16.7)	0	1 (8.3)
Cough	0	1 (16.7)	0	1 (8.3)
Investigations		0	1 (16.7)	1 (8.3)
Transaminases increased	0	0	1 (16.7)	1 (8.3)
Skin and subcutaneous tissue disorders	1 (25.0)	0	0	0
Skin injury	1 (25.0)	0	0	0

a All multiple doses were administered after fasting.

b 0.1 mg was administered on Day 1.

Most TEAEs were mild in severity (four participants experienced severe TEAEs) and straightforward to manage. Four participants received concomitant medications: paracetamol, antacid tablets, ibuprofen, and triamcinolone. One serious TEAE was observed – a severe case of bradycardia associated with transient asystole, approximately 1 hour after receiving icanbelimod 2.5 mg during the single-dosing phase. The participant’s heart rate dropped from 55 to 33 bpm and, 10 hours later, he experienced asystole for 9 seconds; after overnight observation, the participant recovered spontaneously without sequelae. The other three severe TEAEs were lymphopenia (<0.5x10^9^ cells/L), reported for three participants after treatment with icanbelimod 0.25 mg QD; in each instance, lymphocyte counts returned to baseline levels one week after the last dose.

Two participants discontinued the study due to non-serious TEAEs, both after receiving 0.25 mg icanbelimod QD (**Supplementary Figure 1**). One participant discontinued after an increase above the normal upper range in alanine transaminase (129 U/L), without significant changes in other liver enzymes or bilirubin; the participant’s alanine transaminase (64 U/L), bilirubin, and lipid levels were elevated at baseline. The other participant, who withdrew after experiencing second-degree AV block, had an abnormal ECG at baseline – prolonged PR interval with slow heart rate.

In each dosing phase, most TEAEs were experienced by 1 participant after receiving icanbelimod (**Tables 2**, **3**). After single dosing, TEAEs reported for ≥2 participants across all four icanbelimod doses were headache (n=6, 28.6%), dizziness (n=4, 19.0%), and transient bradycardia (n=3, 14.3%). Cardiac disorders (sinus bradycardia [n=2], bradycardia [n=1] and transient asystole [n=1]) were reported in participants receiving the highest single dose of icanbelimod 2.5 mg. After multiple dosing, TEAEs reported for ≥2 participants across the two icanbelimod doses (0.15 mg and 0.25 mg QD) were headache (n=6, 50.0%), lymphopenia (n=5, 41.7%), fatigue (n=2, 16.7%), nausea (n=2, 16.7%), and musculoskeletal pain (n=2, 16.7%); one case of sinus bradycardia was observed with multiple dosing of icanbelimod (0.15 mg QD).

**Figure 4:**
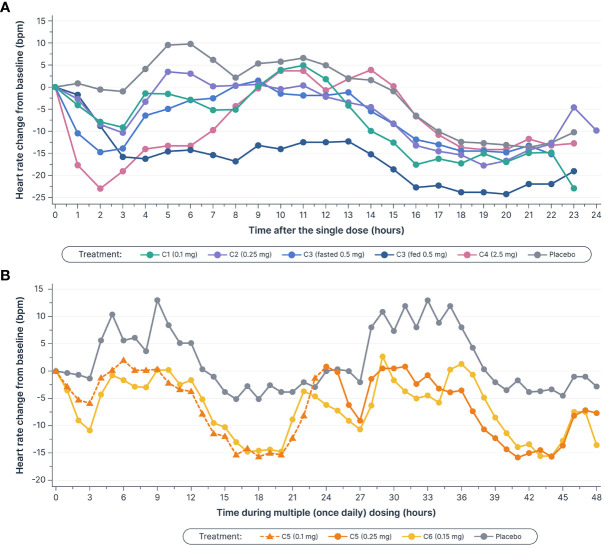
Mean heart rate decreased **(A)** after single doses of icanbelimod in Cohorts 1–4^a–c^ and **(B)**, in Cohort 5, the decreases during the initial 3 hours after 0.1 mg and 0.25 mg doses (0–3 and 24–27 hours, respectively) were attenuated by up-titration, relative to decreases observed with the 0.15 mg once daily dose in Cohort 6^c–f^. ^a^In Cohorts 1 (C1), 2 (C2), and 3 (C3), participants were randomized to single dose icanbelimod 0.1 mg, 0.25 mg, and 0.5 mg (n=6), respectively, or placebo (n=2). ^b^In Cohort 4 (C4), participants were randomized to single dose icanbelimod 2.5 mg (n=4) or placebo (n=1). ^c^All treatments were administered after fasting, except for C3 participants who received a 0.5 mg dose after fasting and returned after one week of washout for an additional 0.5 mg dose administered following a high-fat breakfast. ^d^In Cohort 5 (C5), participants were randomized to icanbelimod 0.1 mg for one day, followed by 0.25 mg once daily (n=6), or placebo (n=2). ^e^In Cohort 6 (C6), participants were randomized to icanbelimod 0.15 mg once daily (n=6) or placebo (n=2). ^f^Administration of icanbelimod 0.1 mg on Day 1, followed on subsequent days by icanbelimod 0.25 mg once daily in C5, was associated with less pronounced reduction in heart rate during the first 2 days of dosing than following administration of icanbelimod 0.15 mg once daily in C6.

Heart rate was monitored continuously for 24 hours in the single-dose phase, and for 24 hours following the first and second doses (48 hours in total) in the multiple-dose phase (**Figure 4**). In the single-dose phase, maximal mean heart rate reduction from baseline within 6 hours post-dose with icanbelimod (0.1, 0.25, 0.5, and 2.5 mg) was 9.0, 10.3, 14.8, and 23.0 bpm, respectively, compared with 1.1 bpm with placebo. In the multiple-dose phase, maximal mean heart rate reduction at 3 hours post-dose on Day 1 was 6.0 bpm (icanbelimod 0.1 mg QD) and 10.8 bpm (icanbelimod 0.15 mg QD), compared with 1.3 bpm with placebo. After the second dose, administered on Day 2, maximal mean heart rate reduction at 3 hours post-dose was 9.1 bpm (following icanbelimod 0.25 mg; these participants received 0.1 mg on Day 1), 10.7 bpm (icanbelimod 0.15 mg QD), and 2.0 bpm with placebo.

## 4 Discussion

In this first-in-human study, icanbelimod oral administration led to dose-dependent reductions in blood lymphocyte counts, which recovered within one week after cessation of treatment, and was generally well tolerated as single and multiple daily dosing regimen by healthy men in Australia. The most common TEAEs were headache, dizziness, fatigue, nausea, and musculoskeletal pain. Most TEAEs were mild in severity. Three participants experienced transient bradycardia, with one adjudged to be serious, following administration of the highest single dose of icanbelimod (2.5 mg), and one case of sinus bradycardia in the multiple-dose cohorts, during treatment with icanbelimod 0.15 mg QD. Icanbelimod was readily absorbed after oral administration and, as with the reduction in lymphocyte counts, exposure increased dose-dependently. Treatment in the fed versus fasted state (investigated after single-dose 0.5 mg icanbelimod) may increase exposure and moderately improve PD effects, i.e. reduction in lymphocyte counts. In subsequent studies, icanbelimod has only been administered in the fed state (another completed Phase 1 study in healthy Chinese men and a Phase 2 trial in patients with moderate-to-severe UC).

S1PR modulators, which activate G protein-gated inwardly rectifying potassium channels on atrial myocytes, are associated with bradycardia and second-degree AV block (30). As expected, icanbelimod was associated with a transient reduction in heart rate. Previous studies have demonstrated that a dose-titration regimen of the S1PR_1_ agonist, ponesimod, can attenuate the observed heart rate reduction (31). In the current study, administration of icanbelimod 0.1 mg on Day 1, followed on subsequent days by icanbelimod 0.25 mg QD, was associated with less pronounced reduction in overall heart rate during the first 2 days of dosing than following administration of icanbelimod 0.15 mg QD. These results suggest that a starting dose of 0.1 mg or less may be appropriate as an initial titration dose in future clinical studies of icanbelimod.

Icanbelimod was readily absorbed with a mean T_max_ of 5.9 hours and an elimination half-life of 25.2 hours, both shorter than the first-in-class S1PR modulator fingolimod (T_max_, 12 hours; half-life, ~192 hours) (17). The PK characteristics of icanbelimod were generally similar to other next-generation S1PR modulators, including ponesimod and siponimod (26, 27, 30, 31). However, when indirectly comparing clinical trials, caution should be applied owing to potential differences in study design and conduct, including sample size and characteristics, which may impact findings.

Icanbelimod demonstrated high potency in reducing circulating lymphocyte counts, with maximum decreases of 49% and 75% with 0.15 mg and 0.25 mg QD dosing, respectively, on Day 14. This was similar to other S1PR modulators including fingolimod (70–80%), amiselimod (32–68%), and ponesimod (approximately 75%) (17, 25, 26, 32–36) and is likely to be related to PK characteristics such as half-life (29). Clinical studies of fingolimod have shown that lymphocyte counts can remain depressed several weeks after discontinuation of treatment (17, 18). This prolonged suppression of lymphocyte count correlates with the long elimination half-life of fingolimod. Other S1PR agonists also demonstrate prolonged reductions in lymphocyte counts that, for instance, may not fully recover until >48 days after ceasing amiselimod treatment (15 days for ozanimod, and 14 days for siponimod), although recovery to within the normal range (i.e. still below baseline levels) was substantially quicker for all three medications (25, 27, 30). In comparison, icanbelimod demonstrated recovery of lymphocyte counts to baseline levels within one week following the last treatment. Fast recovery of lymphocyte counts may be beneficial to patients, protecting against opportunistic infection from occurring with reduced immune responses.

This first-in-human study is limited by population size (N=44), who were generally Caucasian and all participants were male. The results are compatible with those of another Phase 1 trial of icanbelimod in healthy Han Chinese men (N=30), particularly regarding reduction in lymphocyte count, their post-treatment recovery, and potential attenuation of the effects on heart rate with titration. Similarly, published studies of ponesimod or fingolimod demonstrated no notable PD differences according to ethnicity (Japanese versus Caucasian) or sex (35, 37, 38). However, given that fingolimod may cause fetal harm (39, 40), only men were enrolled in the two Phase 1 trials of icanbelimod.

In summary, orally administered icanbelimod was effective at reducing lymphocyte counts in healthy men, which recovered within one week after cessation of treatment, and was generally well-tolerated in this Phase 1 trial. These results support evaluation of icanbelimod in patients with autoimmune diseases. Icanbelimod is being investigated in a Phase 2 trial of patients with moderate-to-severe UC.

## Data availability statement

The original contributions presented in the study are included in the article/**Supplementary Material**. Further inquiries can be directed to the corresponding author/s.

## Ethics statement

The studies involving humans were approved by Independent Ethics Committee at Alfred Hospital (Melbourne, Australia). The studies were conducted in accordance with the local legislation and institutional requirements. The participants provided their written informed consent to participate in this study.

## Author contributions

JL: Investigation, Writing – original draft, Writing – review & editing. XY: Conceptualization, Formal Analysis, Resources, Writing – original draft, Writing – review & editing. JG: Conceptualization, Formal Analysis, Resources, Writing – original draft, Writing – review & editing. WP: Conceptualization, Formal Analysis, Resources, Writing – original draft, Writing – review & editing. ZW: Conceptualization, Formal Analysis, Resources, Writing – original draft, Writing – review & editing.

## References

[1] Almutairi K Nossent J Preen D Keen H Inderjeeth C. The global prevalence of rheumatoid arthritis: a meta-analysis based on a systematic review. Rheumatol Int. (2021) 41:863–77. doi: 10.1007/s00296-020-04731-0 33175207

[2] Alinaghi F Tekin HG Burisch J Wu JJ Thyssen JP Egeberg A. Global prevalence and bidirectional association between psoriasis and inflammatory bowel disease-A systematic review and meta-analysis. J Crohns Colitis. (2020) 14:351–60. doi: 10.1093/ecco-jcc/jjz152 31504363

[3] Conrad N Misra S Verbakel JY Verbeke G Molenberghs G Taylor PN. Incidence, prevalence, and co-occurrence of autoimmune disorders over time and by age, sex, and socioeconomic status: a population-based cohort study of 22 million individuals in the UK. Lancet. (2023) 401:1878–90. doi: 10.1016/S0140-6736(23)00457-9 37156255

[4] Dahlhamer JM Zammitti EP Ward BW Wheaton AG Croft JB. Prevalence of inflammatory bowel disease among adults aged ≥18 years — United states, 2015. MMWR Morb Mortal Wkly Rep. (2016) 65:1166–9. doi: 10.15585/mmwr.mm6542a3 27787492

[5] Alatab S Sepanlou SG Ikuta K Vahedi H Bisignano C Safiri S. The global, regional, and national burden of inflammatory bowel disease in 195 countries and territories, 1990–2017: a systematic analysis for the Global Burden of Disease Study 2017. Lancet Gastroenterol Hepatol. (2020) 5:17–30. doi: 10.1016/S2468-1253(19)30333-4 31648971 PMC7026709

[6] Le Berre C Honap S Peyrin-Biroulet L. Ulcerative colitis. Lancet. (2023) 402:571–84. doi: 10.1016/S0140-6736(23)00966-2 37573077

[7] Chotiyarnwong P McCloskey EV. Pathogenesis of glucocorticoid-induced osteoporosis and options for treatment. Nat Rev Endocrinol. (2020) 16:437–47. doi: 10.1038/s41574-020-0341-0 32286516

[8] Bruscoli S Febo M Riccardi C Migliorati G. Glucocorticoid therapy in inflammatory bowel disease: mechanisms and clinical practice. Front Immunol. (2021) 12:691480. doi: 10.3389/fimmu.2021.691480 34149734 PMC8209469

[9] Kandiel A. Increased risk of lymphoma among inflammatory bowel disease patients treated with azathioprine and 6-mercaptopurine. Gut. (2005) 54:1121–5. doi: 10.1136/gut.2004.049460 PMC1774897 16009685

[10] Li J-X Cummins CL. Fresh insights into glucocorticoid-induced diabetes mellitus and new therapeutic directions. Nat Rev Endocrinol. (2022) 18:540–57. doi: 10.1038/s41574-022-00683-6 PMC9116713 35585199

[11] Xie X Li F Chen J-W Wang J. Risk of tuberculosis infection in anti-TNF-α biological therapy: From bench to bedside. J Microbiol Immunol Infection. (2014) 47:268–74. doi: 10.1016/j.jmii.2013.03.005 23727394

[12] Leone GM Mangano K Petralia MC Nicoletti F Fagone P. Past, present and (Foreseeable) future of biological anti-TNF alpha therapy. JCM. (2023) 12:1630. doi: 10.3390/jcm12041630 36836166 PMC9963154

[13] Mao-Draayer Y Sarazin J Fox D Schiopu E. The sphingosine-1-phosphate receptor: A novel therapeutic target for multiple sclerosis and other autoimmune diseases. Clin Immunol. (2017) 175:10–5. doi: 10.1016/j.clim.2016.11.008 PMC5315594 27890706

[14] Chaudhry BZ Cohen JA Conway DS. Sphingosine 1-phosphate receptor modulators for the treatment of multiple sclerosis. Neurotherapeutics. (2017) 14:859–73. doi: 10.1007/s13311-017-0565-4 PMC5722770 28812220

[15] Chun J Giovannoni G Hunter SF. Sphingosine 1-phosphate receptor modulator therapy for multiple sclerosis: differential downstream receptor signalling and clinical profile effects. Drugs. (2021) 81:207–31. doi: 10.1007/s40265-020-01431-8 PMC7932974 33289881

[16] Jozefczuk E Guzik TJ Siedlinski M. Significance of sphingosine-1-phosphate in cardiovascular physiology and pathology. Pharmacol Res. (2020) 156:104793. doi: 10.1016/j.phrs.2020.104793 32278039

[17] Kovarik JM Schmouder R Barilla D Riviere G-J Wang Y Hunt T. Multiple-dose FTY720: tolerability, pharmacokinetics, and lymphocyte responses in healthy subjects. J Clin Pharmacol. (2004) 44:532–7. doi: 10.1177/0091270004264165 15102874

[18] Francis G Kappos L O’Connor P Collins W Tang D Mercier F. Temporal profile of lymphocyte counts and relationship with infections with fingolimod therapy. Mult Scler. (2014) 20:471–80. doi: 10.1177/1352458513500551 23950550

[19] Arvin AM Wolinsky JS Kappos L Morris MI Reder AT Tornatore C. Varicella-zoster virus infections in patients treated with fingolimod: risk assessment and consensus recommendations for management. JAMA Neurol. (2015) 72:31. doi: 10.1001/jamaneurol.2014.3065 25419615 PMC5391035

[20] Redelman-Sidi G Michielin O Cervera C Ribi C Aguado JM Fernández-Ruiz M. ESCMID Study Group for Infections in Compromised Hosts (ESGICH) Consensus Document on the safety of targeted and biological therapies: an infectious diseases perspective (Immune checkpoint inhibitors, cell adhesion inhibitors, sphingosine-1-phosphate receptor modulators and proteasome inhibitors). Clin Microbiol Infection. (2018) 24:S95–107. doi: 10.1016/j.cmi.2018.01.030 PMC5971148 29427804

[21] Selmaj K Li DK Hartung H-P Hemmer B Kappos L Freedman MS. Siponimod for patients with relapsing-remitting multiple sclerosis (BOLD): an adaptive, dose-ranging, randomised, phase 2 study. Lancet Neurol. (2013) 12:756–67. doi: 10.1016/S1474-4422(13)70102-9 23764350

[22] Bravo GÁ Cedeño RR Casadevall MP Ramió-Torrentà L. Sphingosine-1-phosphate (S1P) and S1P signaling pathway modulators, from current insights to future perspectives. Cells. (2022) 11:2058. doi: 10.3390/cells11132058 35805142 PMC9265592

[23] Cohen JA Comi G Selmaj KW Bar-Or A Arnold DL Steinman L. Safety and efficacy of ozanimod versus interferon beta-1a in relapsing multiple sclerosis (RADIANCE): a multicentre, randomised, 24-month, phase 3 trial. Lancet Neurol. (2019) 18:1021–33. doi: 10.1016/S1474-4422(19)30238-8 31492652

[24] Kappos L Fox RJ Burcklen M Freedman MS Havrdová EK Hennessy B. Ponesimod compared with teriflunomide in patients with relapsing multiple sclerosis in the active-comparator phase 3 OPTIMUM study: A randomized clinical trial. JAMA Neurol. (2021) 78:558. doi: 10.1001/jamaneurol.2021.0405 33779698 PMC8008435

[25] Harada T Wilbraham D de la Borderie G Inoue S Bush J Camm AJ. Cardiac effects of amiselimod compared with fingolimod and placebo: results of a randomised, parallel-group, phase I study in healthy subjects. Brit J Clin Pharma. (2017) 83:1011–27. doi: 10.1111/bcp.13203 PMC5401982 27921320

[26] Sugahara K Maeda Y Shimano K Mogami A Kataoka H Ogawa K. Amiselimod, a novel sphingosine 1-phosphate receptor-1 modulator, has potent therapeutic efficacy for autoimmune diseases, with low bradycardia risk: Amiselimod: pharmacology and effects on heart rate. Br J Pharmacol. (2017) 174:15–27. doi: 10.1111/bph.13641 27714763 PMC5221453

[27] Tran JQ Hartung JP Peach RJ Boehm MF Rosen H Smith H. Results from the first-in-human study with ozanimod, a novel, selective sphingosine-1-phosphate receptor modulator. J Clin Pharmacol. (2017) 57:988–96. doi: 10.1002/jcph.887 PMC5516232 28398597

[28] Meno-Tetang GML Li H Mis S Pyszczynski N Heining P Lowe P. Physiologically based pharmacokinetic modeling of FTY720 (2-amino-2[2-(-4-octylphenyl)ethyl]propane-1,3-diol hydrochloride) in rats after oral and intravenous doses. Drug Metab Dispos. (2006) 34:1480–7. doi: 10.1124/dmd.105.009001 16751263

[29] Scott FL Clemons B Brooks J Brahmachary E Powell R Dedman H. Ozanimod (RPC1063) is a potent sphingosine-1-phosphate receptor-1 (S1P1) and receptor-5 (S1P5) agonist with autoimmune disease-modifying activity. Br J Pharmacol. (2016) 173:1778–92. doi: 10.1111/bph.13476 PMC4867749 26990079

[30] Gergely P Nuesslein-Hildesheim B Guerini D Brinkmann V Traebert M Bruns C. The selective sphingosine 1-phosphate receptor modulator BAF312 redirects lymphocyte distribution and has species-specific effects on heart rate. Br J Pharmacol. (2012) 167:1035–47. doi: 10.1111/j.1476-5381.2012.02061.x PMC3485666 22646698

[31] Brossard P Scherz M Halabi A Maatouk H Krause A Dingemanse J. Multiple-dose tolerability, pharmacokinetics, and pharmacodynamics of ponesimod, an S1P _1_ receptor modulator: Favorable impact of dose up-titration: The Journal of Clinical Pharmacology. J Clin Pharmacol. (2014) 54:179–88. doi: 10.1002/jcph.244 24408162

[32] D’Ambrosio D Steinmann J Brossard P Dingemanse J. Differential effects of ponesimod, a selective S1P _1_ receptor modulator, on blood-circulating human T cell subpopulations. Immunopharmacol Immunotoxicol. (2015) 37:103–9. doi: 10.3109/08923973.2014.993084 25519470

[33] Jurcevic S Juif P-E Hamid C Greenlaw R D’Ambrosio D Dingemanse J. Effects of multiple-dose ponesimod, a selective S1P_1_ receptor modulator, on lymphocyte subsets in healthy humans. DDDT. (2016) 11:123–31. doi: 10.2147/DDDT.S120399 PMC5207338 28096659

[34] Brossard P Derendorf H Xu J Maatouk H Halabi A Dingemanse J. Pharmacokinetics and pharmacodynamics of ponesimod, a selective S1P1 receptor modulator, in the first-in-human study. Br J Clin Pharmacol. (2013) 76:888–96. doi: 10.1111/bcp.12129 PMC3845312 23594176

[35] Kovarik JM Slade A Voss B Schmidli H Riviere GJ Picard F. Ethnic sensitivity study of fingolimod in white and Asian subjects. CP. (2007) 45:98–109. doi: 10.5414/CPP45098 17323789

[36] Choden T Cohen NA Rubin DT. Sphingosine-1 phosphate receptor modulators: the next wave of oral therapies in inflammatory bowel disease. Gastroenterol Hepatol (N Y). (2022) 18:265–71. PMC9666818 36397756

[37] Reyes M Hoch M Brossard P Dingemanse J. Effects of ethnicity and sex on the pharmacokinetics and pharmacodynamics of the selective sphingosine-1-phosphate receptor 1 modulator ponesimod: A clinical study in Japanese and caucasian subjects. Pharmacology. (2014) 94:223–9. doi: 10.1159/000368837 25402365

[38] Hoch M D’Ambrosio D Wilbraham D Brossard P Dingemanse J. Clinical pharmacology of ponesimod, a selective S1P_1_ receptor modulator, after uptitration to supratherapeutic doses in healthy subjects. Eur J Pharm Sci. (2014) 63:147–53. doi: 10.1016/j.ejps.2014.07.005 25046167

[39] Pauliat E Onken M Weber-Schoendorfer C Rousson V Addor M-C Baud D. Pregnancy outcome following first-trimester exposure to fingolimod: A collaborative ENTIS study. Mult Scler. (2021) 27:475–8. doi: 10.1177/1352458520929628 32538681

[40] GILENYA (fingolimod) FDA label . Available online at: https://www.accessdata.fda.gov/drugsatfda_docs/label/2018/022527s024lbl.pdf.

