# Supplementary material for: Icanbelimod (CBP-307), a next-generation Sphingosine-1-phosphate receptor modulator, in healthy men: pharmacokinetics, pharmacodynamics, safety, and tolerability in a randomized trial in Australia
Source: Front Immunol. 2024 Jun 17;15:1380975. doi: 10.3389/fimmu.2024.1380975 (PMC11216006; doi:10.3389/fimmu.2024.1380975)
Supplement: Supplementary file 1 [file DataSheet_1.docx]

**Contents**

[**Supplementary Table 1.** Inclusion and exclusion criteria 2](#_Toc165561802)

[**Supplementary Table 2.** PK and PD collection timepoints 4](#_Toc165561803)

[**Supplementary Table 3.** Baseline demographics 5](#_Toc165561804)

[**Supplementary Figure 1.** Study completion 6](#_Toc165561805)

# **Supplementary Table 1.** Inclusion and exclusion criteria

| **Inclusion criteria** |
| --- |
| Participants meeting all of the following criteria were considered for enrollment into the study:  1) Healthy male subjects age between 18 and 55 years  2) BMI between 19 and 30 kg/m^2^  3) No clinically significant findings in the medical history and physical examination, especially with regard to the liver and gastrointestinal systems  4) No clinically significant laboratory values and urinalysis, unless the investigator considers any abnormality to be clinically irrelevant  5) Normal ECG, blood pressure, and heart rate, unless the investigator considers any abnormality to be clinically irrelevant  6) FEV_1_ within normal range at screening or on Day -1  7) Signed Informed Consent |
| **Exclusion criteria** |
| Participants presenting with any of the following were excluded in the study:  1) Family history of premature Coronary Heart Disease  2) QTcB or QTcF ≥ 450 msec  3) AST, ALT, alkaline phosphatase and bilirubin greater than 1.5xULN (isolated bilirubin >1.5xULN is acceptable if bilirubin is fractionated and direct bilirubin <35%)  4) History of uveitis  5) Subjects with a history of, or examination suspicious for, skin cancer(s) including melanoma, basal cell or squamous cell carcinoma  6) Any condition requiring the regular use of any medication  7) Exposure to prescription medications or to drugs known to interfere with metabolism of drugs within 30 days prior to screening  8) Exposure to any other medication, including over-the counter medications, herbal remedies and vitamins 14 days prior to randomization except paracetamol  9) Participation in another study with any investigational drug in the 2 months preceding the study  10) Treatment in the previous 3 months with any drug known to have a well-defined potential for toxicity to a major organ  11) Positive urine cotinine result at screening or on Day -1  12) Be in the exclusion period of any previous study with investigational drugs  13) Symptoms of a clinically significant illness in the 3 months before the study  14) Presence or sequelae of gastrointestinal, liver or kidney disease, or other conditions known to interfere with the absorption, distribution, metabolism, or excretion of drugs  15) Chronic constipation or diarrhea, irritable bowel syndrome, inflammatory bowel disease  16) Hemorrhoids or anal diseases with regular or recent presence of blood in feces  17) History of significant allergic disease (e.g. medications) and acute phase of allergic rhinitis in the previous 2 weeks before randomization or any food allergy  18) History of Acute or chronic bronchospastic disease (including asthma and chronic obstructive pulmonary disease)  19) Blood or plasma donation of more than 500 mL during the previous 2 month before randomization and/or more than 50 mL in the 2 weeks prior to screening  20) Subjects at risk for TB, specifically subjects with:  21) Current clinical, radiographic or laboratory evidence of active TB  22) History of active TB unless there is documentation that the prior anti-TB treatment was appropriate in duration and type  23) Latent TB which has not been successfully treated  24) A positive quantiFERON^®^ test at screening or within 6 months prior to Day 1  25) Known positive test for HIV  26) Known positive test for hepatitis B (antigens HBs, antibody HBc) or C, unless caused by immunization  27) History of shingles or recurrent episodes of HSV1 or HSV2 infections  28) Current evidence of drug abuse or history of drug abuse within one year before randomization  29) History of alcohol abuse or active alcoholism  30) Mental condition rendering the subject incapable to understand the nature, scope, and possible consequences of the study  31) Adults under guardianship and people with restriction of freedom by administrative or legal decisions  32) Unlikely to comply with the clinical study protocol; e.g. uncooperative attitude, inability to return for follow-up visits, and improbability of completing the study  33) Subject is the investigator or any sub-investigator, research assistant, pharmacist, study coordinator, other staff or relative thereof directly involved in the conduct of the protocol  34) Systolic blood pressure less than 95 mmHg or greater than 140 mmHg, or diastolic blood pressure less than or equal to 50 mmHg or greater than or equal to 95 mmHg  35) Resting heart rate:  a. >90 bpm at screening, or at check-in on Day -1, or at 1h pre-dose on Day 1  b. <60 beats per minute at screening or at check-in on Day -1, or <55 bpm at 1 hour pre-dose on Day 1 for subjects receiving less than 0.5 mg study drug;  c. <65 bpm at screening or at check-in on Day -1, or <60 bpm at 1h pre-dose on Day 1 for subjects receiving ≥0.5 mg study drug |

ALT, alanine aminotransferase; AST, aspartate aminotransferase; BMI, body mass index; bpm, beats per minute; ECG, electrocardiogram; FEV_1_, Forced Expiratory Volume in 1 second; HIV, human immunodeficiency virus; HSV, herpes simplex virus; QTcB, Bazett's Correction Formula; QTcF, Fridericia's Correction Formula; TB, tuberculosis; ULN, upper limit of normal.

# **Supplementary Table 2.** PK and PD collection timepoints

| **Single dosing** | |
| --- | --- |
| **Day** | **Timepoint** |
| **1** | 10 min pre-dose, 30 min, 1h, 2h, 4h, 6h, 8h, 10h, 12h post-dose |
| **2** | 24h, 36h |
| **3** | 48h |
| **4** | 72h |
| **Multiple dosing** | |
| **1** | 10 min pre-dose, 30 min, 1h, 2h, 4h, 6h, 8h, 10h, 12h post-dose |
| **2** | 10 min pre-dose |
| **3** | 10 min pre-dose |
| **7** | 10 min pre-dose |
| **14** | 10 min pre-dose |
| **21** | 10 min pre-dose |
| **28** | 10 min pre-dose, 30 min, 1h, 2h, 4h, 6h, 8h, 10h, 12h post-dose |
| **29** | 24h after Day 28 dosing |
| **30** | 48h after Day 28 dosing |
| **35** | 168h after Day 28 dosing (PD collection only) |
| **42** | 336h after Day 28 dosing (PD collection only) |

PD, pharmacodynamics; PK, pharmacokinetics.

# **Supplementary Table 3.** Baseline demographics

|  | **Placebo**  N=11 | **Icanbelimod**  N=33 | **Total**  N=44 |
| --- | --- | --- | --- |
| **Age** (years) | 28.1  ± 5.56 | 25.2  ± 4.87 | 25.9  ± 5.14 |
| **Height** (cm) | 180.1  ± 7.65 | 177.9  ± 8.16 | 178.5  ± 8.00 |
| **Weight** (kg) | 76.4  ± 6.37 | 75.8  ± 8.58 | 76.0  ± 8.02 |
| **BMI** (kg/m^2^) | 23.6  ± 1.87 | 24.0  ± 2.36 | 23.9  ± 2.24 |

All values are mean ± standard deviation. Most participants were Caucasian; two were Asian, two were other ethnic origin.

BMI, body mass index

# **Supplementary Figure 1.** Study completion

# **
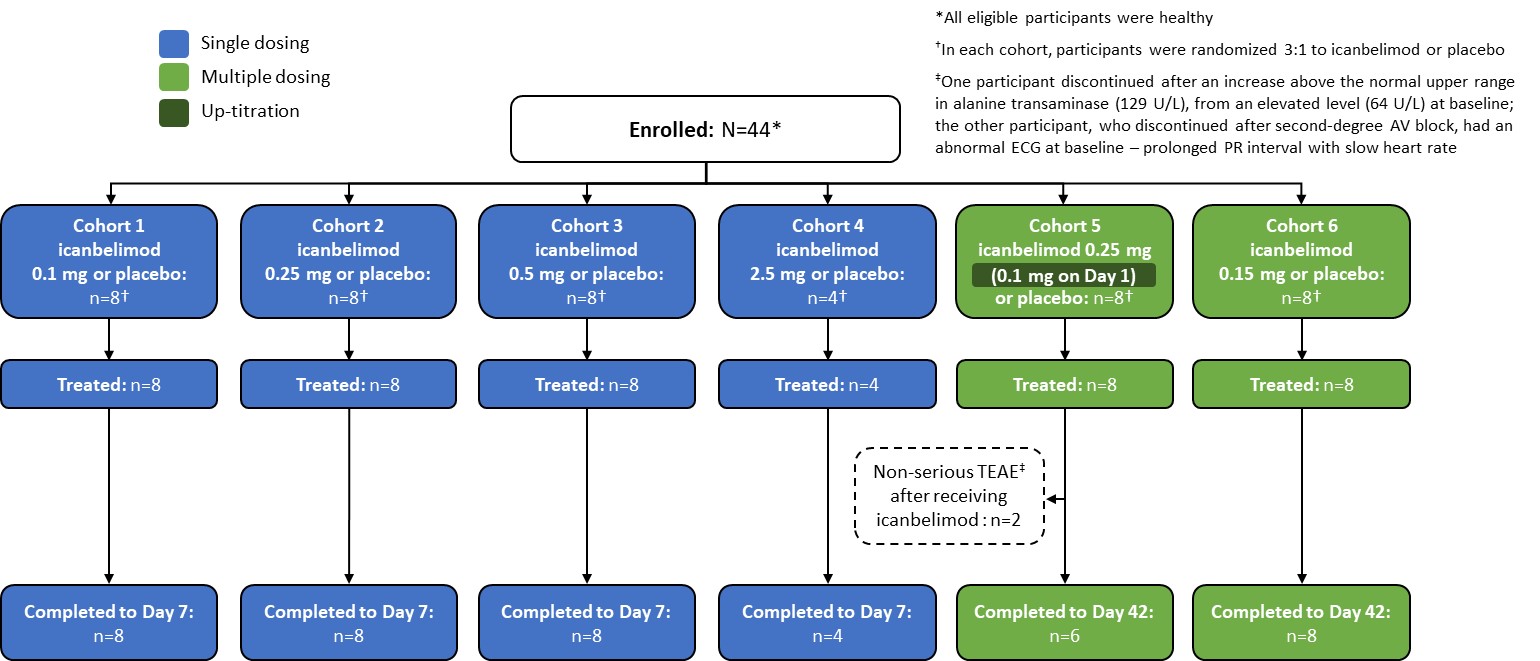
**
